# Investigating Neuroimaging Correlates of Early Frailty in Patients With Behavioral Variant Frontotemporal Dementia: A MRI and FDG-PET Study

**DOI:** 10.3389/fnagi.2021.637796

**Published:** 2021-04-14

**Authors:** Martina Amanzio, Sara Palermo, Mario Stanziano, Federico D'Agata, Antonello Galati, Salvatore Gentile, Giancarlo Castellano, Massimo Bartoli, Giuseppina Elena Cipriani, Elisa Rubino, Paolo Fonio, Innocenzo Rainero

**Affiliations:** ^1^Department of Psychology, University of Turin, Turin, Italy; ^2^European Innovation Partnership on Active and Healthy Ageing (EIP-AHA), Brussels, Belgium; ^3^Centro Interdipartimentale di Studi Avanzati in Neuroscienze - National Institute of Turin (NIT), Orbassano, Italy; ^4^Neuroradiology Unit, Fondazione IRCCS Istituto Neurologico “Carlo Besta,” Milan, Italy; ^5^Department of Neuroscience “Rita Levi Montalcini,” University of Turin, Turin, Italy; ^6^Neuroradiology Unit, Department of Neuroscience “Rita Levi Montalcini,” University of Turin, Turin, Italy; ^7^Nuclear Medicine, Azienda Ospedaliera Universitaria “Città della Salute e della Scienza di Torino,” Turin, Italy; ^8^Aging Brain and Memory Clinic, Neurology I, Department of Neuroscience “Rita Levi Montalcini,” University of Turin, Turin, Italy; ^9^Department of Diagnostic Imaging and Radiotherapy, Radiology Institute, Azienda Ospedaliera Universitaria “Città della Salute e della Scienza di Torino,” University of Turin, Turin, Italy

**Keywords:** behavioral variant frontotemporal dementia, frailty, magnetic resonance imaging, voxel-based morphometry, positron emission tomography

## Abstract

Frailty is a dynamic clinical condition characterized by the reduction of interconnections among different psychobiological domains, which leads to a homeostatic vulnerability. The association between physical frailty and cognitive dysfunctions is a possible predictor of poor prognosis in patients with neurodegenerative disorders. However, this construct has not been fully analyzed by a multidimensional neuropsychogeriatric assessment matched with multimodal neuroimaging methods in patients with behavioral variant frontotemporal dementia (bvFTD). We have investigated cognitive dysfunctions and frailty status, assessed by both a neuropsychological evaluation and the Multidimensional Prognostic Index (MPI), in a sample of 18 bvFTD patients and compared to matched healthy controls. Gray matter (GM) volume (as assessed by voxel-based morphometry) and metabolism (on ^18^fluorodeoxyglucose positron emission tomography) were first separately compared between groups, then voxelwise compared and correlated to each other within patients. Linear regression of the MPI was performed on those voxels presenting a significant correlation between altered GM volume and metabolism. The neuropsychological assessment reflected the diagnoses and the functional–anatomical alterations documented by neuroimaging analyses. In particular, the majority of patients presented significant executive dysfunction and mood changes in terms of apathy, depression, and anxiety. In the overall MPI score, the patients fell in the lower range (indicating an early frailty status). On imaging, they exhibited a bilateral decrease of GM density and hypometabolism involving the frontal pole, the anterior opercular region, and the anterior cingulate cortex. Greater atrophy than hypometabolism was observed in the bilateral orbitofrontal cortex, the triangular part of the inferior frontal gyrus, and the ventral striatum, whereas the contrary was detected in the bilateral dorsal anterior cingulate cortex and pre-supplementary motor area. MPI scores significantly correlated only with the co-occurrence of a decrease of GM density and hypometabolism in the right anterior insular cortex, but not with the separate pathological phenomena. Our results show a correlation between a specific pattern of co-occurring GM atrophy and hypometabolism with early frailty in bvFTD patients. These aspects, combined with executive dysfunction and mood changes, may lead to an increased risk of poor prognosis, highlighting a potentially critical and precocious role of the insula in the pathogenesis of frailty.

## Introduction

Frailty is an age-related dynamic status characterized by a reduced resistance to stressors due to the cumulative decline of multiple neuropsychophysiological systems (Bartoli et al., 2020). Currently, the concept of frailty is considered as a potential antecedent of age-related diseases (Alonso et al., 2014). This construct has triggered growing attention in many medical areas (Hoogendijk et al., 2019) as it likely contributes to the relevant variability of health outcomes. Furthermore, it may affect the health trajectories of individuals presenting similar risk profiles (e.g., diagnosed with the same disease) (Rockwood and Howlett, 2019).

Three main models to study frailty in aging subjects are currently used. The “phenotypic” (Fried et al., 2001) and “deficit accumulation” models (Rockwood et al., 2005; Rockwood and Mitnitski, 2007) characterize the biomedical approach. It highlights how a reduction in the ability to preserve physiological homeostasis and to respond to environmental changes appropriately implies a loss of functional autonomy (Xue, 2011). Specifically, the phenotypic model (Fried et al., 2001) considers frailty in terms of a physiopathological syndrome composed of a decrease in hand grip strength and in physical activity, unintentional weight loss, asthenia, and a decrease in gait speed. The authors identify a frailty condition with the presence of three or more criteria and a pre-frailty status with the presence of one or two criteria. The deficit accumulation model (Rockwood et al., 2005; Rockwood and Mitnitski, 2007) provides a frailty index (FI), a continuous variable that is obtained by quantifying the set of age-related deficits that configure a vulnerability increased by age-related decline in various body organs and physiological systems (Rockwood and Howlett, 2019).

FI scores may be associated with a progressive neurocognitive disorder in patients with mild cognitive impairment (MCI) and with negative prognostic outcomes in Alzheimer's disease (AD) subjects (Canevelli et al., 2020). Rockwood et al. (2007) pointed out that the deficit accumulation model allows a more comprehensive assessment of frailty and is more sensitive to negative prognostic outcomes than the phenotypic model (Fried et al., 2001). However, the authors did not consider all the factors involved in the complex phenomenon of frailty from a multidimensional perspective, in line with the biopsychosocial model (Gobbens et al., 2010).

Importantly, a multidimensional approach has been emphasized to better understand frailty, not only as a pathophysiological syndrome (Canevelli et al., 2015) but also in association with a general cognitive decline, jointly considering the role of psychosocial factors among the determinants of this critical status (Matusik et al., 2012). In line with this approach, the Multidimensional Prognostic Index (MPI) could be considered a more comprehensive evaluation tool, useful for the assessment of subjects with neurodegenerative disorders, from minor to major neurocognitive decline, with different frailty status (Pilotto et al., 2020). A recent study has suggested that cognitive dysfunctions, mood changes, and a reduction in the instrumental activities of daily living have a significant role in the neuropsychological aspects associated with frailty in patients with neurodegenerative disorders. In particular, a significant association between frailty—as measured by the MPI—and metacognitive–executive dysfunction, depression, and disinhibition has been outlined in MCI likely due to AD and mild AD. These results were specific and not influenced by other cognitive functions such as global cognition, memory, language comprehension, and non-verbal reasoning (Amanzio et al., 2017).

Despite considerable literature on frailty in community-dwelling subjects, only studies on AD patients investigated disease biomarkers associated with a frailty status. In particular, Wallace et al. (2018) conducted a scoping review to investigate such association, considering postmortem AD pathology, brain atrophy, and *in vivo* fluid markers. The authors showed that, in 80% of the analyzed studies, a higher degree of frailty was associated with at least one biomarker abnormality. The same authors investigated the role of frailty, in terms of FI, in the relationship between neuropathology and clinical expression of dementia (Wallace et al., 2019). Their findings indicated that a low level of frailty seemed to allow a better tolerance of AD pathology and, consequently, a lower clinical expression of the disorder, whereas high levels of frailty seemed to be related to both greater AD pathology and worse cognitive impairment (Wallace et al., 2019).

Canevelli et al. (2020) described how clinical manifestation influences the association between pathological changes due to AD and cognitive decline. The authors found that subjects with higher FI scores had lower cerebrospinal fluid (CSF) levels of amyloid beta 1–42 (Aβ1–42), hippocampal volumes on MRI, and glucose metabolism on fluorodeoxyglucose positron emission tomography (FDG-PET) and greater amyloid deposition on ^18^F-AV-45 (Florbetapir F-18) PET. In addition, they showed both a strengthened relationship between dementia with FDG-PET and a weakened relationship between dementia and ^18^F-AV-45 uptake, and hippocampal volume was related to worsening frailty status.

Taking these studies into account, it can be hypothesized that the individual's frailty status may modify the association between AD biomarkers and the cognitive manifestations occurring along the AD continuum (Jack et al., 2011). Frailty emerges as a reduction of reserves, which enables the organism and the brain to tolerate the onset of pathological perturbations and modifications with limited functional consequences. Moreover, frailty is characterized by some biological processes, such as metabolic declines and inflammation, which may intervene in the onset of a neurocognitive disorder (Bisset and Howlett, 2019).

To the best of our knowledge, no previous studies have investigated the correlates of frailty in patients with behavioral variant frontotemporal dementia (bvFTD), i.e., by means of a comprehensive *multidimensional* neuropsychogeriatric assessment and *multimodal* neuroimaging techniques.

In view of all the above, we hypothesized and sought to investigate a potential relationship between a frailty status in patients with bvFTD and the presence of possible early disease-specific structure functional cerebral changes. To address this issue, we directly correlated brain *structural* (MRI) and *metabolic* (^18^fluorodeoxyglucose PET, ^18^FDG-PET) imaging modalities with each other and with the MPI in a dataset of bvFTD patients. Specifically, we aimed to investigate gray matter volumetric and metabolic modifications together with the regional variations of their reciprocal hierarchy and to correlate the imaging results with a well-validated clinical prognostic score of frailty (Pilotto et al., 2008, 2009; Volpato et al., 2015), also associated with possible metacognitive dysfunctions and mood changes, in line with the results obtained in subjects with MCI likely due to AD and with AD (Amanzio et al., 2017). Specifically, our major endpoints were: (1) to investigate gray matter volumetric and metabolic modifications together with the regional variations of their reciprocal hierarchy; (2) to correlate the imaging results with MPI as a well-validated clinical prognostic score of frailty (Pilotto et al., 2008, 2009; Volpato et al., 2015); and (3) to describe specific cognitive dysfunctions and mood changes.

## Materials and Methods

### Participants

Participants complaining of cognitive impairment were admitted as inpatients to the Aging Brain and Memory Clinic of the Department of Neuroscience of the University of Turin (Italy) and were investigated according to a standardized protocol. The diagnosis was performed according to the International Behavioral Variant Frontotemporal Dementia Criteria Consortium (FTDC) (Rascovsky et al., 2011). Patients underwent extensive clinical, genetic, neuropsychological, and neuroradiological investigations, the latter including both brain ^18^FDG-PET and high-resolution structural MRI. Patients were excluded from the study if they had a history of neurological and psychiatric disorders other than bvFTD and if their structural imaging revealed lesions due to stroke, traumatic injury, brain tumor, or inflammatory diseases. All patients underwent lumbar puncture with a CSF measurement of 1–42 beta-amyloid, phospho-tau, and total tau (Innogenetics kits, Ghent, Belgium) in order to exclude AD pathology. No patients with a movement disorder in association with cognitive abnormalities were enrolled in the study.

Cognitively preserved subjects living in the same geographical area, with normal neurological and psychiatric evaluations, were enrolled as age- and sex-matched controls for neuroimaging analyses.

Written informed consent was obtained from all participants. The study was approved by the Hospital Ethics Committee and was in accordance with the latest version of the Declaration of Helsinki.

### Frailty Measurement

Considering the importance of tools respecting clinimetric criteria (De Vries et al., 2011), frailty was evaluated using the MPI, which derives from a standardized comprehensive geriatric assessment (CGA) (Pilotto et al., 2008, 2013). It can be considered an instrument for the assessment of the multidimensionality of frailty (Pilotto et al., 2020) as it examines cognitive, functional, biological, and social aspects (Giordano et al., 2007; Pilotto et al., 2007).

The MPI provides a prognostic index of mortality in the short- (1 month) and long-term (1 year) periods based on information obtained in hospitalized and outpatient settings (Volpato et al., 2015). Specifically, MPI is characterized by eight domains: (1) activity of daily living (ADL); (2) instrumental activity of daily living (IADL); (3) Short Portable Mental Status Questionnaire (SPMSQ); (4) Cumulative Illness Rating Scale—Comorbidity Index (CIRS-CI); (5) Mini Nutritional Assessment (MNA, to assess the nutritional status); (6) Exton Smith Scale (ESS, to evaluate the risk of developing pressure sores); (7) polypharmacy; and (8) social condition. The obtained numerical index reports three grades of prognostic risk of mortality: low, moderate, and severe. MPI effectiveness has been recently verified in population-based cohorts (Maggi and Pilotto, 2017). Higher MPI risk scores are associated with longer hospitalization and fewer years of survival across a broad and stratified age range (Angleman et al., 2015; Volpato et al., 2015). The MPI is also considered suitable for assessing frailty in the elderly (Giordano et al., 2007; Pilotto et al., 2007, 2008). In particular, it supports the identification of a frailty status in elders and permits individual interventions, thus reducing medications and hospitalizations (Maggi and Pilotto, 2017).

To verify whether matched controls presented aspects of physical frailty, the following phenotypic model variables were considered: handgrip strength reduction, gait speed reduction, physical activity reduction, asthenia, and weight loss (Fried et al., 2001).

### Neuropsychological Investigations

The neuropsychological evaluation included an assessment of global cognitive impairment using the Addenbrooke's Cognitive Examination—revised version (ACE-R) (Mioshi et al., 2006), which also provides the Mini Mental State Examination (MMSE) score (Folstein et al., 1975). Two other cognitive screening tests were used in order to assess executive functions: the Montreal Cognitive Assessment (MoCA) (Conti et al., 2015) and the Frontal Assessment Battery (FAB) (Appollonio et al., 2005). Moreover, specific cognitive domains were assessed by the Attentional Matrices for selective attention (Spinnler and Tognoni, 1987), trail making test (TMT) for divided attention and cognitive shifting (Spinnler and Tognoni, 1987; Reitan and Wolfson, 1994), recall of a short story and Rey memory test—immediate recall—for episodic memory (Babcock) (Spinnler and Tognoni, 1987), Colored Progressive Matrices (CPM-36) for reasoning in the visual modality (Spinnler and Tognoni, 1987), Token test (TT) for language comprehension (Spinnler and Tognoni, 1987), and the phonemic verbal fluency (Spinnler and Tognoni, 1987). The instruments used to assess executive functions were the Behavioral Assessment of Dysexecutive Syndrome (BADS) (Wilson et al., 1996) and the metacognitive version of the Wisconsin card sorting test (m-WCST) for executive functions (Koren et al., 2006).

This version of the test differs from the original as subjects are asked two questions to assess the “on-line” metacognitive monitoring (“What is your degree of confidence in this answer?”) and control (“Do you want to take this response into account in your total score?”) for each card.

In this study, patients received a monetary gain of 10 cents for each correct answer, and they were deprived of 10 cents in the case of a wrong answer. A set of indices, described in our previous study (Amanzio et al., 2014) were evaluated: (1) accuracy score (AS), the number of correct voluntary answers/number of voluntary responses; (2) free choice improvement (FCI) (accuracy – number of correct responses from forced responses)/number of cards presented; (3) global monitoring (GM), the number of correct responses – the total number of sorts required in the final score; (4) monitoring resolution (MR), the gamma correlation calculated between the confidence and correctness of the sorts in the entire test; (5) control sensitivity (CS), to what extent the control process depended on the monitoring process, indexed by the gamma correlation calculated across all sorts between the level of confidence and the decision to gamble; (6) monetary gains (MG), given by the number of correct voluntary responses – incorrect number of voluntary responses (Amanzio et al., 2014, p. 138).

Patients were also assessed using neuropsychiatric rating scales of mood changes: apathy and depression with the Hamilton Depression Rating scale (HDR-S) (Hamilton, 1960), anxiety with the Hamilton Rating Scale for Anxiety (Hamilton, 1959), disinhibition and hypomania with the Disinhibition Scale (DIS-S) (Starkstein et al., 2004), and the Mania Scale (MAS) (Bech et al., 1978).

Matched controls were assessed by MMSE in order to exclude subjects with global cognitive impairment.

### Genetic and Cerebrospinal Fluid Investigations

All bvFTD patients were screened according to standardized methods for mutations in the progranulin (PGRN) and microtubule-associated protein tau (MAPT) genes and genotyped for the apolipoprotein E (APOE) alleles and expansions in the *C9orf72* gene. CSF examination was performed in all bvFTD patients, thus excluding any comorbidity. In addition to the standard examination, the CSF concentrations of beta-amyloid (Aβ1–42), total tau (t-Tau), and 181-phospho-tau (p-Tau) were determined using ELISA kits (Innogenetics, Ghent, Belgium).

### Procedures

Patients were neuropsychologically assessed in three sessions, for about 1 h on different days, in order to avoid mental and physical tiredness. Moreover, they underwent neuroimaging evaluation before the lumbar puncture for CSF tests.

### Imaging Protocols, Parameters, and Analysis

#### PET Scans

PET studies were performed at the Nuclear Medicine Department, AOU of “Città della Salute e della Scienza” Hospital in Turin, Italy, on a Philips Gemini scanner (Philips Medical System, Cleveland, OH, USA) the day before the MRI examination. Reconstructed brain images had a dimension of 128 × 128 × 90 voxels (2 mm^3^ × 2 mm^3^ × 2 mm^3^). After the planned whole-body ^18^FDG-PET/CT examination was performed, the coronal, sagittal, and transverse datasets were reconstructed using a 3D iterative technique (row action maximum likelihood algorithm, RAMLA-3D) and corrected with single scatter simulation (SSS).

#### MRI Structural Scans

MRI scans were performed at AOU of “Città della Salute e della Scienza” Hospital, Department of Neuroscience, Turin, Italy. The structural brain MRI scans of all participants were acquired on a 1.5-T MRI scanner (Achieva, Philips). T1-weighted 3D Turbo Field Echo (TFE) sequences [matrix = 256 × 256, voxel size = 1 mm^3^ × 1 mm^3^ × 1 mm^3^, number of slices = 190, repetition time (TR) = 7 ms, echo time (TE) = 3 ms, TFE shots = 89] were obtained with full brain coverage and isotropic voxel equivalent to an MPRAGE (magnetic prepared rapid acquisition gradient echo).

#### Image Preprocessing, Analyses, and Visualization

In part, we replicated the processing pipeline described and employed by Buhour et al. (2017), which was specifically designed to provide a direct voxelwise comparison of the degree of local gray matter atrophy and hypometabolism (Pitel et al., 2009) while controlling for partial volume effects (PVEs) in atrophic brains.

Data preprocessing and analyses were performed using the Statistical Parametric Mapping software package (SPM12; “Wellcome” Center for Human Neuroimaging, London; http://www.fil.ion.ucl.ac.uk/spm) running on MATLAB 7.5 (MathWorks, Natick, MA, USA). Images were overlaid with MRIcron software (http://people.cas.sc.edu/rorden/mricron/index.html) on the Montreal Neurological Institute (MNI) template brain. To obtain anatomical localization of significant cluster peaks, the Automated Anatomical Labeling (AAL) and the Brodmann area (BA) templates were used.

#### Preprocessing of Metabolic Data

The ^18^FDG-PET data were PVE corrected using the modified Müller–Gärtner method (Müller-Gärtner et al., 1992) implemented in the PVElab software package (Quarantelli et al., 2004), co-registered onto their corresponding MRI scans, spatially normalized using the DARTEL toolbox in order to obtain a high-dimensional normalization protocol (Ashburner, 2007), and smoothed with a Gaussian kernel of 8-mm full width at half maximum (FWHM). In order to remove confounding effects of global activity differences, the count of each voxel to the mean count of a standardized pontine region of interest (ROI) was normalized to avoid biased normalization. The ROI was a rectangular multislice region (*x*/*x*′ = −8/8, *y*/*y*′ = −32/−24, *z*/*z*′ = −44/−34; MNI space), sampling 144 voxels on the central pons, and manually drawn on the PET SPM template using the MRIcro application (http://www.sph.sc.edu/comd/rorden/mricro.html). A previous careful visual inspection of the pons was conducted on each spatially normalized but non-smoothed brain scan in order to detect metabolic changes, which could alter the ROI measure. Then, the same ROI was used on each spatial normalized and smoothed brain image and the pons mean voxel values (*Y*_p_) sampled. Using the image calculation tool of SPM, the scaled voxel values (*Y*′) of each brain was set at *Y*′ = (*Y*/*Y*_p_), where *Y* is the non-scaled (“raw”) voxel value. Only voxel values >80% of the cerebral metabolic rate of glucose consumption (CMRglc) were included in the analysis.

#### Preprocessing of Structural Data

The MRI data were preprocessed using the VBM8 toolbox (http://dbm.neuro.uni-jena.de/vbm.html) with default parameters incorporating the Diffeomorphic Anatomical Registration using Exponentiated Lie algebra (DARTEL) (Ashburner, 2007). The images were bias corrected, tissue classified, and registered by using linear (12-parameter affine) and nonlinear transformations (warping) within a unified model (Ashburner and Friston, 2000), affine transformed into the MNI space and scaled by the Jacobian determinants of the deformations to account for the local compression and stretching occurring as a consequence of the warping and affine transformation (modulation), and smoothed with a Gaussian kernel of 8-mm FWHM (resulting in a smoothness identical to that of PET images) to allow a direct comparison between the structural and metabolic GM values (Buhour et al., 2017). The total intracranial volume (TICV) was also estimated as the sum of CSF, white matter (WM), and GM.

#### Masking of Imaging Modalities

A binary GM mask obtained from the union of the GM of the two study sample groups (patients plus controls), as described in Villain et al. (2008), was applied to both PET and MR individual images to prevent voxel misclassification and include only GM voxels of interest in the following statistical comparisons.

#### Between-Group Comparisons of the GM Density of Tissue and Metabolism

The two study sample groups were first compared independently within each imaging modality using the general linear model (GLM) on a voxel-by-voxel basis throughout the whole brain to obtain separate maps of significant GM atrophy and hypometabolism in bvFTD patients compared with matched controls. TICV was entered as a covariate of no interest. Significant results were identified using a family-wise error (FWE) threshold of *p* < 0.05, with a minimum cluster size of 160 and 40 voxels automatically calculated for structural and metabolic images, respectively.

#### Voxelwise Correlation Between the GM Density of Tissue and Hypometabolism

Patients' individual *z*-score maps for each voxel and for each modality were created by dividing the difference between patients' individual values and controls' mean by the controls' standard deviation (Buhour et al., 2017). Then, patients' individual *z*-score maps of the two different modalities were entered pairwise in a voxelwise correlation analysis using the BPM toolbox of SPM (Casanova et al., 2007), which showed all the voxels presenting a linear correlation between the GM density of tissue and hypometabolism in patients. The results were thresholded at *p* < 0.05, FWE corrected for multiple comparisons with a minimum cluster size of 150 voxels automatically calculated.

#### Within-Group Comparison Between the Degree of Structural and Metabolic Abnormalities

The FDG uptake and the GM density of tissue *z*-score maps were averaged across all subjects, one-tail thresholded at *p* < 0.05 (corresponding to a minimum *z*-score value of −2), transformed into two masks to restrict the comparison of each modality only within those voxels with significant values of GM density of tissue or hypometabolism, and then applied to patients' individual *z*-score maps. The “masked” *z*-score maps of each patient were entered in a one-tailed *t*-test analysis with one group (patient) and two imaging modalities per subject. The resulting contrasts (FDG uptake *z*-scores vs. the GM density of tissue *z*-scores) yielded statistical maps reflecting any predominance of atrophy over hypometabolism and *vice versa* in patient group. The results were thresholded at *p* < 0.05, FWE corrected for multiple comparisons with a minimum cluster size of 150 voxels for both contrasts.

#### Voxelwise Correlation Between Frailty, Atrophy, and Hypometabolism

A voxelwise regression of the MPI (adjusted for TICV, age, and time since diagnosis) was performed within patient group on those voxels presenting a linear correlation between density of tissue and hypometabolism or any predominance of density of tissue over hypometabolism (and *vice versa*). Since the study aimed to investigate early clinical signs of frailty, then the main effects of bvFTD had to be ruled out. Indeed, disease severity has been included as a covariate in the analyses. Significant effects were identified using *p* < 0.05, FWE corrected with a minimum cluster size of 150 voxels.

#### Other Statistical Comparisons

All comparisons of demographic and neurobehavioral measures were conducted with the Statistical Package for Social Sciences (SPSS) 13.0 software package. Where appropriate, differences in the demographic, clinical, and neuropsychological data were assessed using parametric and nonparametric tests. In *post-hoc* analyses, Tukey's test and the Mann–Whitney *U*-test were employed for ANOVA and Kruskal–Wallis tests, respectively. For categorical measures, χ^2^ tests were applied. For each test statistic, a probability value <0.05 was regarded as significant.

## Results

Over a 24-month period, 64 patients were screened. Of these, 13 fulfilled the criteria for *probable* bvFTD (male/female = 5/8) and five fulfilled the criteria for *possible* bvFTD (male/female = 2/3), in accordance with Rascovsky et al. (2011). Genetic testing and CSF examination did not reveal any mutation, comorbidity, or alternative neurodegenerative diagnosis other than bvFTD (see Supplementary Table 1). Therefore, the final study sample consisted of 18 (seven males, 11 females) patients with a clinically consistent diagnosis of bvFTD and as many cognitively unimpaired controls matched for age, gender, and years of education (detailed demographic characteristics are reported in Table 1). All controls had no neuropsychological impairment (as evaluated by MMSE) or history of neurological and psychiatric disorders. Finally, all controls were classifiable as robust as none of them had reported the presence of any single physical frailty determinant (Fried et al., 2001).

**Table 1:** Synopsis of the final study sample characteristics.

	**bvFTD patients**	**Cognitively unimpaired controls**	**Group difference (*p*)**
No. of subjects	18	18	1
Age, mean ± SD (years)	69.47 ± 9.29	68.22 ± 6.87	0.7^a^
Gender (M/F)	7/11	7/11	1^b^
Education (years)	8.96 ± 3.94	9.02 ± 2.74	1^a^
MMSE	22.39 ± 1.4	28.65 ± 0.4	<0.001^a^
**Time since diagnosis (months)**		
Mean ± SD	23.44 ± 12.45		
Minimum	6		
Maximum	48	–	–

*SD, standard deviation; M, male; F, female; bvFTD, behavioral variant frontotemporal dementia; MMSE, Mini Mental State Examination*.

a *As tested with one-way ANOVA*.

b *As tested with two-tailed chi-square test*.

### Prevalence of Frailty Status and Neuropsychological Assessment

Considering the CGA evaluation, the overall MPI scores fall in the lowest range (0.23 ± 0.11), attesting to a low risk of severe prognosis. However, issues have been found in some of the MPI domains. In particular, patients had, on average, mild impairments in cognitive functioning (SPMSQ), nutritional status (MNA), and physical health status, as indicated by the number of medications taken by subjects (polypharmacy) and the CIRS-CI score.

The neuropsychological assessment reflected the diagnoses and functional-anatomical alterations documented by neuroimaging (Table 2).

**Table 2:** Evaluation of frailty and neuropsychological characteristics of bvFTD patients.

**Assessment tool**	**Mean ± SD**	**Cutoff**
**Frailty assessment**		
Multidimensional Prognostic Index (MPI)	0.25 ± 0.10	–
Comprehensive geriatric assessment		
Activity of daily living scale	5.55 ± 0.70	≥4
Instrumental activity of daily living scale	6.16 ± 1.54	≥6
Short portable mental state questionnaire	2.77 ± 1.52	≤2
Cumulative illness rating scale—comorbidity index	1.83 ± 1.20	<1
Mini nutritional assessment	20.08 ± 3.75	≥24
Exton smith scale	18.5 ± 1.85	≥15
Polypharmacy	4.66 ± 2.49	≤3
Social condition	Household	–
**Neuropsychological assessment**		
Mini mental statement examination	24.16 ± 2.81	≥23.8
Addenbrooke's cognitive examination—revised version	66.16 ± 13.23	≥79
Clinical dementia rating scale	0.69 ± 0.35	–
Frontal assessment battery	11.61 ± 3.71	≥13.48
Montreal cognitive assessment	16.77 ± 4.39	≥17.36
Attentional matrices	31.33 ± 8.97	≥31
Trail making test A	104.72 ± 54.65	≤94
Trail making test B	328.55 ± 149.19	≤283
Trail making test B-A	223.72 ± 121.51	≤187
Babcock	6.02 ± 3.44	≥4.75
Rey memory test −15 instant words	23.73 ± 13.17	≥28.53
Colored progressive matrices −36	20.38 ± 6.66	≥18.96
Phonemic verbal fluency	20.50 ± 9.79	≥17.35
Token test	28.49 ± 4.55	≥26.5
Wisconsin card sorting test, % correct answers	50.95 ± 15.05	≥37.1
Wisconsin card sorting test, % perseverative errors	33.13 ± 16.35	≤42.7
**Wisconsin card sorting test—metacognitive version**		
Accuracy score	0.01 ± 0.002	–
Free choice improvement	−0.50 ± 0.15	–
Global monitoring	−23.16 ± 11.81	–
Monitoring resolution	0.20 ± 0.21	–
Control sensitivity	0.01 ± 0.52	–
Monetary gains	2.18 ± 1.91	–
Behavioral assessment of dysexecutive syndrome	8.88 ± 3.10	≥15
Hamilton depression rating scale	13.33 ± 10.60	≤7
Hamilton rating scale for anxiety	14.58 ± 10.42	≤14
Disinhibition scale	12.66 ± 7.80	≤16.9
Mania scale	3.77 ± 4.90	≤15

*Wherever there is a normative value, the cutoff scores are given in the statistical normal direction. The values refer to the normative data for healthy controls matched for age and education*.

*SD, standard deviation; bvFTD, behavioral variant frontotemporal dementia*.

In particular, the majority of patients presented executive dysfunctions: all of the subjects fell below the cutoff scores on BADS, ~67% on FAB, and 55% on TMT-B and TMT B-A. Problems also emerged on the m-WCST (about 39% presented deficit scores in relation to perseverative errors and 11% in relation to the correctness of the answers given) and on phonemic verbal fluency (44%).

In addition, deficit scores were found on global cognitive functioning (about 44% on MMSE, 83% on ACE-R, and 55% on MoCA), attention (50% on attentional matrices and 44% on TMT-A), episodic memory (55% on recall of a short story and 33% on Rey memory test—immediate recall), language (about 33% on Token test), and reasoning (about 33% on CPM-36).

Moreover, considering the behavioral scales, the patients presented mood changes in terms of depression (HDR-S = 67%), anxiety (HAR-S = 47%), disinhibition (DIS-S = 22%), and mania (5%).

### Neuroimaging Assessment

**Figure 1:**
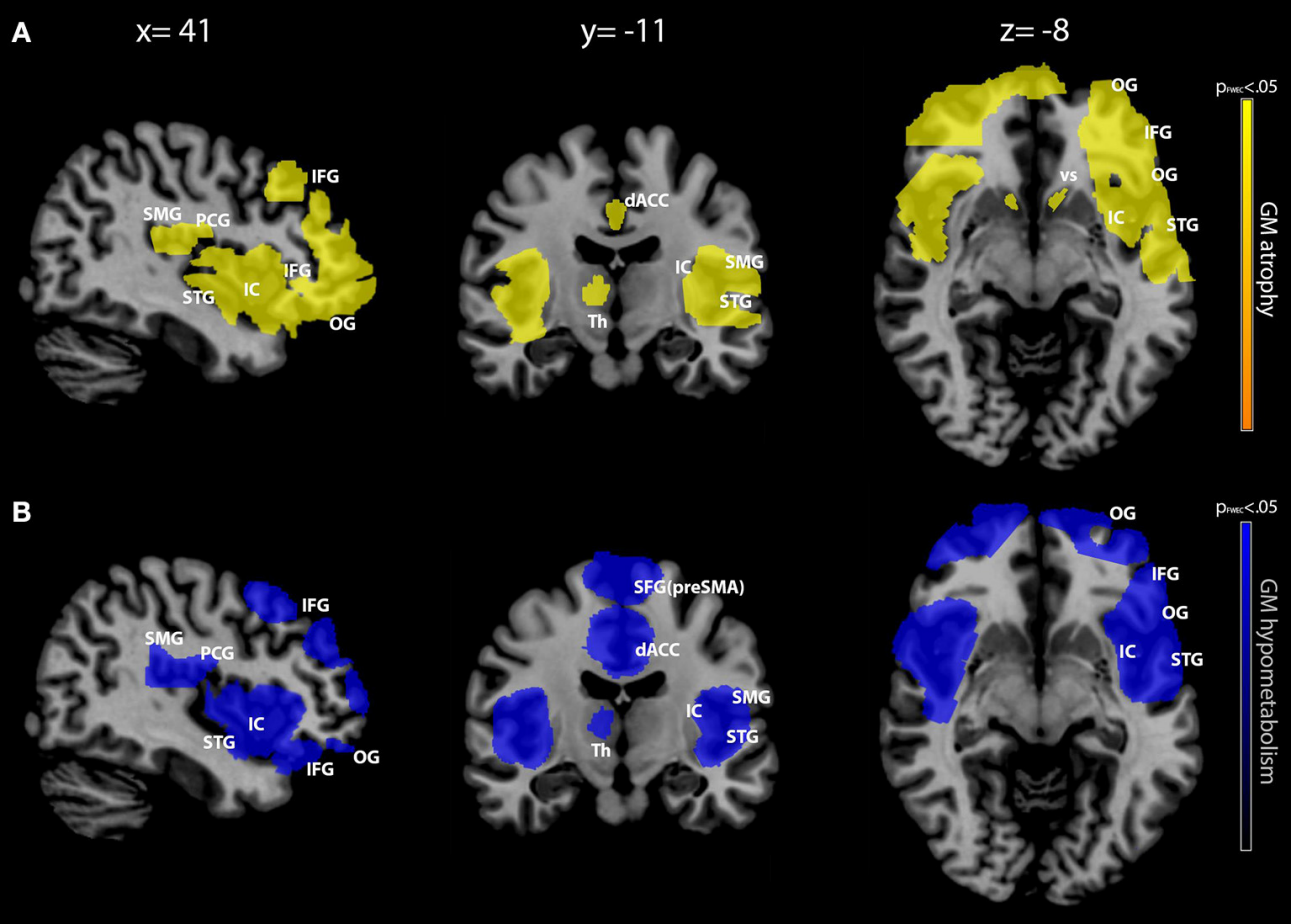
Between-group comparison (patients vs. controls) of gray matter (GM) density and metabolism. GM density [in *yellow, row* **(A)**] and metabolism [in *blue, row* **(B)**] modifications in patients with behavioral variant frontotemporal dementia (bvFTD) compared with cognitively unimpaired controls. Between-group comparisons were conducted separately for each imaging modality with two-sample *t*-test routine. Only clusters surviving a *p* < 0.05 (family-wise error corrected with a minimum cluster size of 160) are shown, as indicated in the *color bars* representing *p*-values on the *left*. Maps are reported in accordance with radiological convention (i.e., left is right). MNI coordinates are on *top of the panel*. OG, orbital gyri; IFG, inferior frontal gyrus; SMG, supramarginal gyrus; PCG, pre-central gyrus; STG, superior temporal gyrus; IC, insular cortex; dACC, dorsal anterior cingulate cortex; SFG (pre-SMA), superior frontal gyrus (pre-supplementary motor area); VS, ventral striatum; Th, thalamus; MNI, Montreal Neurological Institute.

The results for the between-group comparisons (control subjects vs. bvFTD patients for each imaging modality separately) are illustrated in Figure 1 and Table 3. In patients, the spatial patterns of GM density loss and reduced metabolism were similarly distributed, mainly involving bilaterally the opercular region, around and deeply the anterior part of the sulcus lateralis, and the dorsal anterior cingulate cortex (ACC). In more detail, a significant (family-wise error corrected, *p*_FWEC_ < 0.05) GM reduction encompassed the orbital gyri, both the triangular and the dorsal part of the inferior frontal gyrus, the most anterior and lower part of the supramarginal gyrus, the lower end of the pre-central gyrus, the anterior ramus of the superior temporal gyrus, the insular cortex (to a lesser extent), the dorsal part of the ACC, and the right thalamus and bilateral ventral striatum (Figure 1A and Table 3). Significant (*p*_FWEC_ < 0.05) GM hypometabolism was found bilaterally in the dorsal part of the inferior frontal gyrus (and, to a lesser extent, the triangular part of the inferior frontal gyrus and in the orbital gyri), the lower end of the pre-central gyrus, the most anterior and lower part of the supramarginal gyrus, the anterior ramus of the superior temporal gyrus, the insular cortex, the dorsal part of the ACC, the upper dorsal part of the superior frontal gyrus (i.e., pre-supplementary motor area), and the right thalamus (Figure 1 and Table 3).

**Table 3:** Spatial coordinates, extent, and peak values of brain areas showing significant GM density and metabolism changes between groups (bvFTD patients vs. cognitively unimpaired controls).

**Cluster location**	**Ke**	**MNI**			
		***x* (DT/M)**	***y* (DT/M)**	***z* (DT/M)**	***t*, peak value (DT/M)**
R orbital gyri	8,885/4,986	33/21	55/64	−9/−4	7.99/3.25
L orbital gyri	9,387/4,825	−49/−52	31/36	−15/−12	6.65/3.17
R inferior frontal gyrus (triangular part)	12,691/8,222	47/46	23/28	−2/−1	8.32/6.38
L inferior frontal gyrus (triangular part)	13,833/7,947	−37/−44	52/22	16/4	7.91/6.12
R inferior frontal gyrus (dorsal part)	9,445/8,765	38/34	50/38	16/38	6.92/6.25
L inferior frontal gyrus (dorsal part)	9,221/7,019	−47/−52	31/44	36/6	8.13/8.01
R supramarginal gyrus (most anterior and lower part)	2,877/2,911	35/33	−23/−20	22/19	6.29/5.92
L supramarginal gyrus (most anterior and lower part)	2,555/3,001	−46/−50	−35/−31	24/15	6.44/6.09
R pre-central gyrus (lower end)	1,443/1,298	48/38	−14/−30	16/19	5.77/4.98
L pre-central gyrus (lower end)	1,999/1,386	−46/−44	−13/−34	17/21	5.27/5.01
R superior temporal gyrus (anterior ramus)	5,391/5,111	47/53	−16/−4	9/2	5.51/5.03
L superior temporal gyrus (anterior ramus)	4,421/4,977	−55/−52	−7/7	5/5	5.11/5.21
R insular cortex	15,445/15,128	41/37	11/20	−1/−5	9.33/9.01
L insular cortex	14,991/15,731	−37/−43	19/2	−2/8	8.32/7.94
R anterior cingulate cortex (dorsal part)	1,991/11,088	1/3	33/30	22/30	3.06/8.11
L anterior cingulate cortex (dorsal part)	1,023/10,934	−5/−3	13/25	41/23	3.95/7.99
R superior frontal gyrus (upper dorsal part)	–/8,882	–/3	–/11	–/57	–/7.02
L superior frontal gyrus (upper dorsal part)	–/7,921	–/−4	–/19	–/49	–/7.33
R ventral striatum	566/–	8/–	11/–	−7/–	3.07/–
L ventral striatum	690/–	−9/–	14/–	−6/–	3.52/–
R thalamus	1,077/1,008	8/4	−16/−15	9/9	3.95/7.99

*L, left; R, right; ke, cluster size in number of voxels; DT/M, density/metabolism; GM, gray matter; bvFTD, behavioral variant frontotemporal dementia; MNI, Montreal Neurological Institute*.

**Figure 2:**
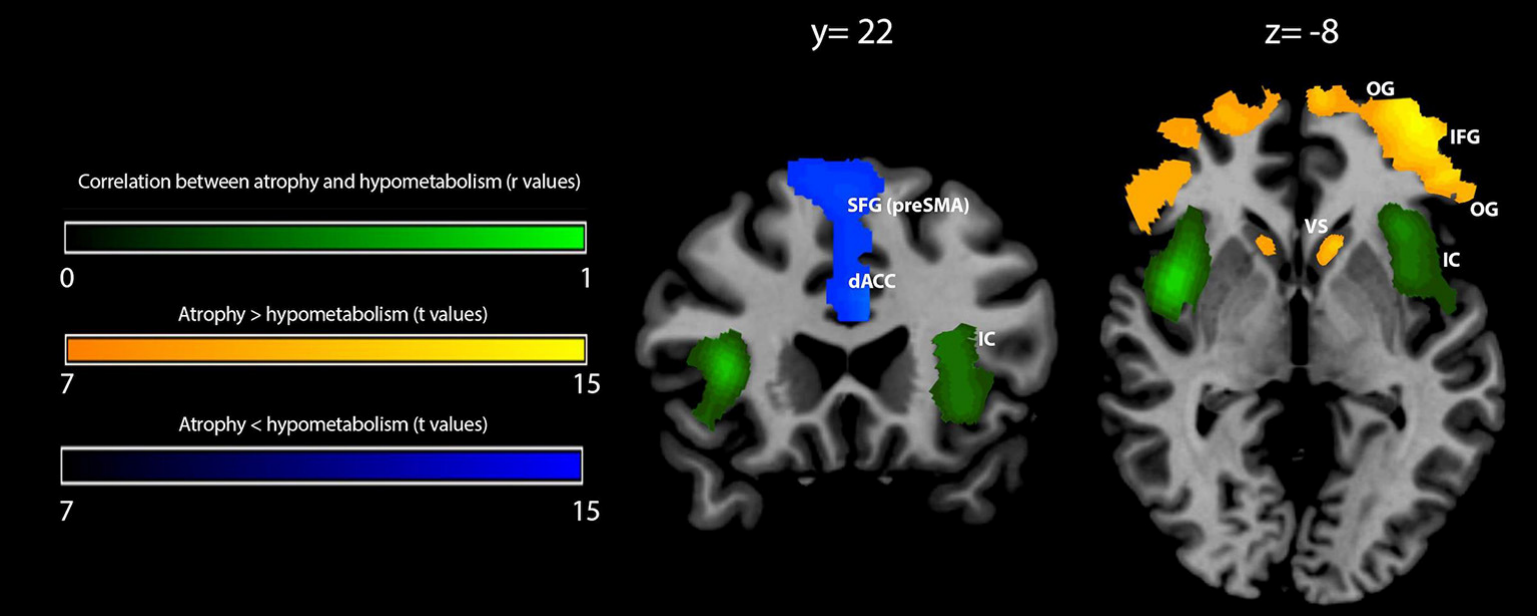
Voxelwise correlation and comparison between gray matter (GM) density loss and hypometabolism in behavioral variant frontotemporal dementia (bvFTD) patients. Correlation between GM density loss and hypometabolism is shown in *green* (color spectrum represents *r*-values of significant voxels); prevalence of density loss over hypometabolism is shown in *orange-yellow*, while the reverse contrast (hypometabolism over atrophy) is shown in *blue* (related color spectra represent *t*-values of significant voxels). Maps are reported in accordance with radiological convention (i.e., left is right). MNI coordinates are on *top of the panel*. OG, orbital gyri; IFG, inferior frontal gyrus; IC, insular cortex; dACC, dorsal anterior cingulate cortex; SFG (pre-SMA), superior frontal gyrus (pre-supplementary motor area); VS, ventral striatum; MNI, Montreal Neurological Institute.

The pairwise intermodality correlation analysis within patient groups (voxelwise correlation between GM density and metabolism) showed significant (*p*_FWEC_ < 0.05) correlations bilaterally in the insular cortex (Figure 2 and Table 4).

**Table 4:** Spatial coordinates, extent, and peak values of areas showing significant correlation (*r*) or prevalence (*t*) between GM density loss and hypometabolism in bvFTD patients.

**Cluster location**	**ke**	**MNI**			
		***x***	***y***	***z***	***r*/*t* peak value**
R insular cortex	9,855	43	13	−2	(*r*) 0.921
L insular cortex	8,992	−41	8	−1	(*r*) 0.905
R orbital gyri	6,599	40	44	−3	(*t*) 10.58
L orbital gyri	7,002	−55	37	−3	(*t*) 15.00
R inferior frontal gyrus (triangular part)	4,398	52	42	2	(*t*) 11.29
L inferior frontal gyrus (triangular part)	8,005	−49	27	3	(*t*) 14.72
R ventral striatum	381	13	23	1	(*t*) 8.11
L ventral striatum	522	−12	19	−3	(*t*) 12.82
R anterior cingulate cortex (dorsal part)	9,016	4	30	21	(*t*) 11.11
L anterior cingulate cortex (dorsal part)	8,901	−6	36	40	(*t*) 12.93
R superior frontal gyrus (upper dorsal part)	9,372	4	6	52	(*t*) 10.28
L superior frontal gyrus (upper dorsal part)	10,019	−5	7	60	(*t*) 10.77

*L, left; R, right; ke, cluster size in number of voxels; GM, gray matter; bvFTD, behavioral variant frontotemporal dementia; MNI, Montreal Neurological Institute*.

Within-group comparison between GM density and metabolism revealed a significant (*p*_FWEC_ < 0.05) prevalence of GM atrophy bilaterally in the orbital gyri, the triangular part of the inferior frontal gyrus, and the ventral striatum. Contrariwise, hypometabolism significantly (*p*_FWEC_ < 0.05) prevailed over atrophy bilaterally in the dorsal part of the anterior cingulate cortex and the upper dorsal part of the superior frontal gyrus (pre-supplementary motor area) (Figure 2 and Table 4).

**Figure 3:**
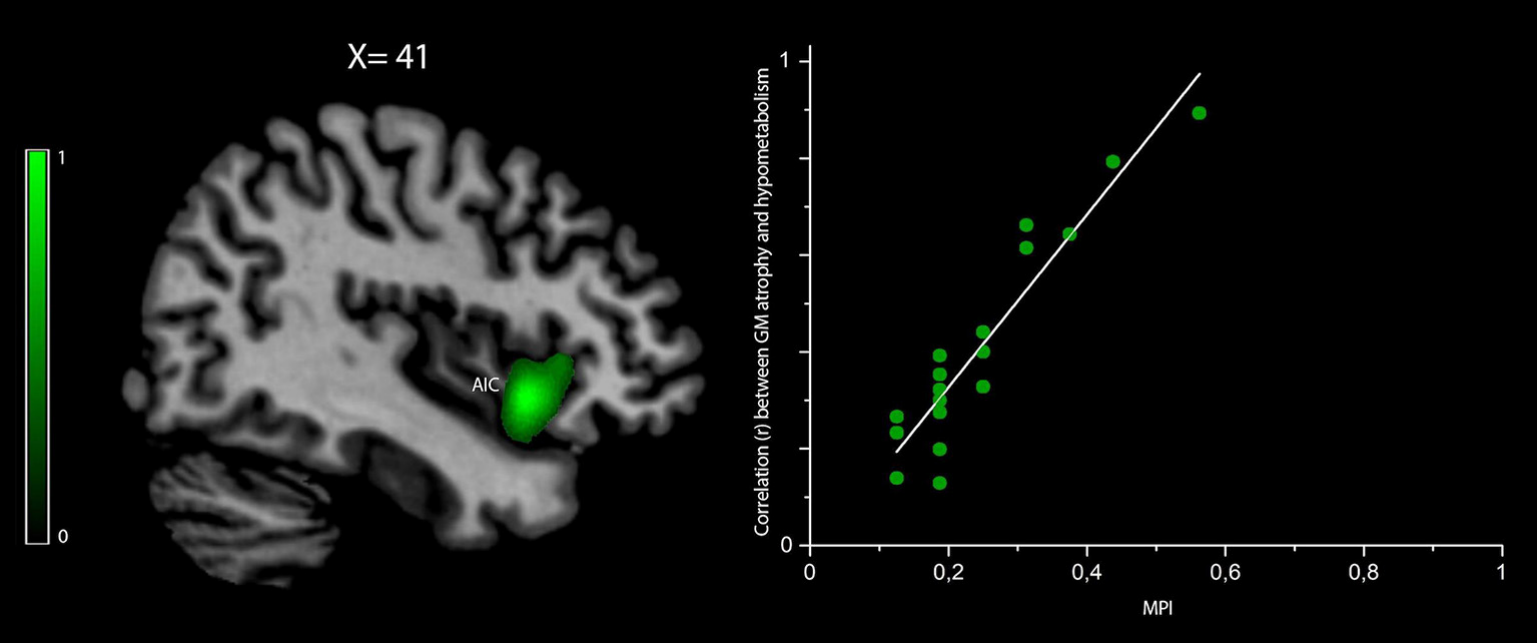
Voxelwise correlation between frailty, density loss, and hypometabolism in behavioral variant frontotemporal dementia (bvFTD) patients. Correlation between the MPI scores and voxels where gray matter (GM) density loss and hypometabolism were linearly correlated (i.e., “co-occurred”) is shown in *green* (color spectrum represents *r*-values). The map is reported in accordance with radiological convention (i.e., left is right). MNI coordinate is on *top of the panel*. AIC, anterior insular cortex; MPI, Multidimensional Prognostic Index; MNI, Montreal Neurological Institute.

The correlation between the MPI values and those voxels where GM density loss and hypometabolism were linearly correlated (i.e., “co-occurred”) was found to be significant (peak *r* = 0.86 at *p*_FWEC_ < 0.05) only in the right anterior insular cortex (cluster size in voxels = 8,943; MNI peak coordinates: *x* = 39, *y* = 19, *z* = −4) (Figure 3). No significant results were obtained for MPI correlation with those voxels showing any predominance of GM density loss vs. hypometabolism or *vice versa*.

## Discussion

To the best of our knowledge, this is the first neuroimaging study investigating neural correlates of *early frailty* in bvFTD, in their initial stage of illness. We examined a *homogeneous* group of bvFTD patients with overall MPI scores that fell in the lowest range (indicating a low risk of poor prognosis) and structuro-metabolic modifications by *multimodal* imaging (structural MRI and ^18^FDG-PET). Specifically, our purpose was to describe GM density and metabolic modifications together with the regional variations of their reciprocal hierarchy and neuropsychological deficits and to correlate individual MPI scores with the location, degree, and reciprocal hierarchy of GM atrophy and hypometabolism.

Patients were compared with a normative group of subjects without physical and cognitive frailty. The thorough investigation of our patients results in considering the complexity of the construct of frailty as a heterogeneous clinical status characterized by the loss of harmonic interactions among various dimensions that lead to a homeostatic instability. This type of accurate analysis is even more important in subjects with initial bvFTD in order to better follow their prognostic pathways. Indeed, and unfortunately, these subjects have a particularly accelerated and unfavorable disease outcome (Eslinger et al., 2005). The importance of our study for the literature is given by the aspects of novelty compared to previous publications: (a) in previous studies, frailty has never been investigated by a multidimensional neuropsychogeriatric assessment combined with multimodal neuroimaging methods in patients with bvFTD; (b) cognitive dysfunctions and mood changes were assessed through an in-depth neuropsychological evaluation; and (c) the diagnosis of bvFTD is supported by accurate investigations, such as CSF analyses in order to exclude AD pathophysiology.

Compared to age-matched cognitively unimpaired robust controls, patients exhibited patterns of GM atrophy and hypometabolism mainly involving bilaterally the ventral prefrontal cortex, the anterior opercular region, and the ACC, as actually expected in bvFTD (Schroeter et al., 2008). Particularly, the brain areas mainly involved were: the orbital gyri, both the triangular and the dorsal part of the inferior frontal gyrus, the most anterior and lower part of the supramarginal gyrus, the lower end of the pre-central gyrus, the anterior ramus of the superior temporal gyrus, the insular cortex, the dorsal part of the ACC, the right thalamus, and bilateral ventral striatum. These findings are in line with previous neuroimaging studies on bvFTD patients, which highlighted the *early* involvement of the fronto-insular transition zones and ACC, while extended atrophy in the posterior hippocampi, head of caudate nucleus, posterior insulae, and the temporal and parietal lobes characterizes bvFTD *advanced* stages (Seeley et al., 2008).

Although our results were expected and quite incremental relative to the existing bvFTD literature, they allowed us to provide a useful “search volume” only composed of those regions typically affected by the disease. Specifically, the purpose of the comparison with healthy controls was to identify the regions of interest for subsequent regression analysis of the MPI, which was intended as the main and original aim of the study, in order to conduct a regression of the MPI values against all voxels within bvFTD-specific regions of interest. As a result, we found a unique correlation of the individual MPI scores with the right anterior insula. Furthermore, our finding showing that the metabolic and structural damages were of equal severity in the insula suggested that frailty, in its early stage, might be associated both with regional hypometabolism and atrophy, without the prevalence of either.

Importantly, the association among MPI, co-occurring atrophy, and hypometabolism was net of patients' severity of disease, age, and months since diagnosis (used as covariates of no interest).

The fact that the metabolic and structural damages in the insula are of equal severity means that the neural correlate of frailty, in its early stage, is associated with both hypometabolism and atrophy. Although the association found is a novel result, because no previous studies in the literature investigated it in bvFTD, nevertheless, this finding does not lead to a definite statement that atrophy of the right anterior insular cortex is a specific sign of early frailty in bvFTD. Reduced gray matter was previously found to be associated with some cortical areas related to executive–metacognitive functions (i.e., the insula, inferior parietal lobule, pre-central gyrus, and anterior cingulate) using whole-brain voxel-based analysis in a frailty-risk group of community-dwelling people (Chen et al., 2015).

Our findings of reduced gray matter in the insula area and associated with an early state of frailty would seem to be in line with Chen's observations (Chen et al., 2015), albeit reported in community-dwelling subjects.

Importantly, the involvement of this specific area may occur in frail subjects suffering from neurodegenerative diseases. For example, in subjects with AD, frailty was associated with global cortical atrophy (Del Brutto et al., 2017; Wallace et al., 2018) and atrophy of specific cortical areas, such as the temporal (Tay et al., 2016), frontal, and peri-insular subcortical region (Gallucci et al., 2018).

In subjects with MCI likely due to AD and in AD patients, from a neuropsychological point of view, an early frailty status may be associated with executive dysfunction as assessed by BADS and m-WCST (Amanzio et al., 2017). In line with these findings, our results suggest the same association in bvFTD patients. In particular, subjects were found to have poor performance on the ability to inhibit a response, self-monitoring, and cognitive set-shifting flexibility [measured by two BADS subtests: rule shift cards (RSC) and modified six elements (MSE)]. Poor performance in self-online monitoring and control was also observed through m-WCST. In addition, a change in mood in terms of apathy–depression, anxiety, and disinhibition was observed. Since executive dysfunction, depression–apathy, and disinhibition seem to be attributable to the malfunction of the same brain network (Masterman and Cummings, 1997; Bonelli and Cummings, 2007), an early frailty status might also be due to a dysfunction of the brain circuits of “top-down” cognitive control mechanisms characterized as early pathological changes in bvFTD. Indeed, these neuropsychological batteries, such as BADS and m-WCST, assess top-down cognitive control and monitoring mechanisms (Amanzio et al., 2013, 2014).

The association between metacognitive dysfunction and an early frailty status is new and suggestive. bvFTD, and its expression of executive dysfunction, is particularly relevant to the study of an early frailty status. Diminished ability to perceive one's own impairments is common in this kind of patients, among whom impaired self-awareness occurs early in their illness and is one of the five core diagnostic features in the Neary criteria (Neary et al., 1998). The clinical characterization of bvFTD patients, i.e., extensive loss of self-monitoring, self-awareness, and self-knowledge (Eslinger et al., 2005), linked to metacognitive dysfunctions associated with medial prefrontal pathophysiology has been supported by studies using “patient vs. informant discrepancy” approaches (Amanzio et al., 2016). This includes how effectively and accurately an individual is able to use self-monitoring and self-knowledge abilities to guide cognition and behavior in social and non-social contexts (Fernandez-Duque et al., 2000). A progressive decline in terms of social behavior (e.g., erratic judgments, disinhibition, and self-monitoring) may be exacerbated by metacognitive deficits (O'Keeffe et al., 2007), also making a prognostic worsening in the presence of a frailty status.

In formulating a theoretically informative and clinically useful model of an early frailty status in bvFTD patients in their initial stage of illness, it is important to consider the independent functioning of the insular cortex, and how this region may interact with more distributed networks, while acknowledging that this process of reverse inference is necessarily speculative. The insula is a complex cerebral region involved in several cognitive, control, and affective functions (Langner et al., 2018). The anterior insular cortex has been identified as a central hub within human emotional awareness, social conduct, and behavioral guidance in terms of executive control (Craig, 2009). While the insula was classically considered a limbic region, evidences from network analysis also suggested a critical role in high-level cognitive control and executive processes, i.e., in response suppression, task switching, and monitoring (Dosenbach et al., 2006). In this direction, the anterior insular cortex (together with the triangular part of the inferior frontal gyrus) and the ACC are jointly activated, consistent with the idea that they cooperate, being part of the same motor and sensory limbic regions, like the motor and somatosensory cortices (Craig, 2009). In particular, the anterior insular cortex was suggested as the probable site for awareness on the basis of its afferent representation of the “feelings” from the body and the ACC as the probable site for the initiation of behaviors (Craig, 2009) and control of directed effort (Weissman et al., 2006). Indeed, the ACC verifies the presence of conflicts from external inputs (Botvinick et al., 2001; Bari and Robbins, 2013) instead of sorting them out (Botvinick et al., 2004; Kerns et al., 2004). As functionally connected to the right anterior insula (Cai et al., 2014), it plays an important role in the system responsible for monitoring significant events (Seeley et al., 2007; Menon and Uddin, 2010). Indeed, if, on the one hand, the right anterior insula is involved in recognizing salient stimuli, on the other hand, the ACC sets up internal attention mechanisms in order to face them (Han et al., 2019).

Previously, we found a relationship in bvFTD patients between reduced awareness of their functional limitation in daily living (IADL) and gray matter reduction referring to the regions mentioned above, which are involved in executive monitoring of voluntary action and processing task-relevant information to avoid interference from irrelevant stimuli (Amanzio et al., 2016). In particular, the dorsal anterior insula and dorsal cingulate cortices are some of the regions identified as major nodes of the task-positive network, which is anticorrelated with the default mode network and seems to be involved in self-referral processes, above all during wakeful rest periods (Fox et al., 2005). These areas may be considered as a “hub” that connects systems involved in metacognitive executive functions such as action monitoring and in the representation of the affective qualities of sensory events and interoceptive signals (Dosenbach et al., 2006). Indeed, action monitoring is particularly important in situations that need higher processing capacity, such as those related to the IADL. It requires a representation of expected values of different actions, as well as the continuous monitoring of outcomes in terms of updating. Action monitoring processes are needed in order to provide feedback and to adapt our behavior to the ongoing situation (MacDonald et al., 2000). Diminished ability to perceive one's own impairments is common in FTD patients, where impaired self-awareness occurs early in their illness (Neary et al., 1998). Self-awareness relies on comparing knowledge of current abilities with past abilities; it may be inaccurate when such knowledge is affected by monitoring deficits in self-referential processes, as in the case of bvFTD patients (Bastin et al., 2012). All these aspects appear to have important relevance to frailty in individuals with bvFTD.

The simultaneous presence of structural and functional alterations, which we observed in our patients, may leave them unable to model the emotional impact of their own physical and cognitive difficulties, such as those seen in the progressive salience network (SN) breakdown (Seeley, 2010). In normal subjects, the SN works to adjust arousal and attention on the basis of perceived relevance of stimuli (Seeley et al., 2008), which in turn relies on the integration and interpretation of homeostatic, affective, motivational, and hedonic signals (Craig, 2009). On the other hand, the SN has been considered selectively vulnerable in prodromal stages of bvFTD (Seeley et al., 2008; Dopper et al., 2013). The relative salience of these signals determines which of them are more likely to capture attention and, consequently, are essential in the processing of information relevant for survival. In this direction, bvFTD patients have shown to experience problems in daily life functioning where they may risk harming themselves or others because they cannot judge situations adequately. Grossman (2002) underlined how these clinical disorders may contribute significantly to their progressive adaptive behavioral difficulties in home, vocational, and social settings, leading to disability and the need for supervisory care (Grossman, 2002; Amanzio et al., 2016). Importantly, the drastic alterations in “socioemotional awareness,” in the earliest stage of disease, are presumed to arise from dysfunctions of the SN insular hub (Toller et al., 2018). However, it remains to be better clarified whether the earliest behavioral symptoms of bvFTD may result from altered functional connectivity preceding structural atrophy (Whitwell et al., 2011) or *vice versa* (Lee et al., 2014), or a concurrent combination of the two mechanisms.

Our study shows, for the first time, a correlation between an early frailty status and the co-occurrence of hypometabolism and decreased GM in the right anterior insula in patients with bvFTD. As neurodegeneration of this area is also observed in other neurodegenerative disorders, future research is needed to understand whether this cortical dysfunction is a specific target of bvFTD in relation to frailty.

### Limitations of the Study

The results of our study must be viewed cautiously and are intended as explorative. The low number of patients examined is its main limitation. However, our patients represent a highly selected population where diagnosis was supported by clinical, neuropsychological, and biochemical investigations. In addition, our patients performed both functional (i.e., metabolic) and structural neuroimaging of the brain, allowing us to correlate behavioral data with imaging abnormalities detected by two different and complementary modalities.

In conclusion, our study sheds light on the possible neural correlates of frailty in its prodromal stages. In particular, we suggest that a better understanding of the frailty mechanisms in bvFTD may rely on insular biology comprehension since this area is associated with individual survival mechanisms. Further researches will be necessary to better clarify the role of the insula, associated with other regions belonging to the SN, which might represent additional biomarkers. These biomarkers should help in tailoring early specific interventions ameliorating patients' adherence to treatments and prognosis.

## Data Availability Statement

The original contributions presented in the study are included in the article/Supplementary Material, further inquiries can be directed to the corresponding author/s.

## Ethics Statement

The studies involving human participants were reviewed and approved by Ethics Committee of the A.O.U. Città della Salute e della Scienza di Torino, Turin, Italy. The patients/participants provided their written informed consent to participate in this study.

## Author Contributions

MA contributed to the conceptualization, validation, resources, methodology, writing the original draft, review and editing, supervision, funding acquisition, and project administration. SP helped with formal analysis, investigation, writing the original draft, and review and editing. MS helped with the methodology, software, formal analysis, investigation, data curation, writing the original draft, and visualization. FD helped with the methodology, software, formal analysis, investigation, data curation, and in writing the original draft. AG, SG, and GC contributed to software, formal analysis, and resources. MB and GEC helped with investigation and review and editing. ER helped with the investigation. PF helped with resources, data curation, and supervision. IR contributed to the conceptualization, validation, resources, writing the original draft, supervision, and funding acquisition. All authors contributed to the article and approved the submitted version.

## Conflict of Interest

The authors declare that the research was conducted in the absence of any commercial or financial relationships that could be construed as a potential conflict of interest.
