# Supplementary material for: Investigating Neuroimaging Correlates of Early Frailty in Patients With Behavioral Variant Frontotemporal Dementia: A MRI and FDG-PET Study
Source: Front Aging Neurosci. 2021 Apr 14;13:637796. doi: 10.3389/fnagi.2021.637796 (PMC8079404; doi:10.3389/fnagi.2021.637796)
Supplement: Supplementary file 1 [file Table_1.docx]

**Investigating Neuroimaging Correlates of Frailty in Patients with behavioral variant Frontotemporal Dementia**

**Supplementary Material**

**Table S1. Cerebrospinal fluid tests synopsis for the overall experimental sample**

| **Patient ID** | **t-tau** | **p-tau** | **Aβ42** |
| --- | --- | --- | --- |
| 01 | 99 | 18 | 774 |
| 02 | 193,4 | 83,3 | 720 |
| 03 | 75 | 28 | 1126 |
| 04 | 75 | 26 | 641 |
| 05 | 75 | 19 | 987 |
| 06 | 627 | 60 | 852 |
| 07 | 75 | 29 | 1215 |
| 08 | 83 | 44 | 679 |
| 09 | 278 | 95 | 663 |
| 10 | 130 | 37 | 609 |
| 11 | 264 | 93 | 716 |
| 12 | 414 | 95 | 707 |
| 13 | 75 | 26 | 1222 |
| 14 | 75 | 27 | 978 |
| 15 | 133 | 42 | 1266 |
| 16 | 75 | 41 | 1101 |
| 17 | * | * | * |
| 18 | 880 | 133 | 679 |

Abbreviations: t-Tau=total-Tau; p-tau=phospho-Tau; Aβ42=beta-amyloid.

Analysis performed using Innogenetics diagnostic kit (Ghent, Belgium).

Normal values: t-Tau: age range 21-50 yrs <300 pg/ml, age range 51-70 yrs: <450 pg/ml, age range >71 yrs: <500 pg/ml; phospho-Tau: <35 pg/ml; Aβ42: >500 pg/ml.

* Anatomical contraindications to cerebrospinal fluid test.
